# ^68^Ga-PSMA PET/CT for Patients with PSA Relapse after Radical Prostatectomy or External Beam Radiotherapy

**DOI:** 10.3390/diagnostics11040622

**Published:** 2021-03-30

**Authors:** Finn Edler von Eyben, Cigdem Soydal, Rie von Eyben

**Affiliations:** 1 Center of Tobacco Control Research, 5230 Odense, Denmark; 2 Department of Nuclear Medicine, Ankara University, 06560 Ankara, Turkey; csoydal@yahoo.com; 3 Department of Radiation Oncology, Stanford University, Stanford, CA 94350, USA; rie.voneyben@gmail.com

**Keywords:** ^68^Ga-PSMA positron emission tomography/computed tomography (PET/CT), non-metastatic prostate cancer, oligometastatic prostate cancer, overall survival (OS), prostate-specific membrane antigen (PSMA), prostate-specific antigen (PSA) relapse, salvage treatment

## Abstract

The study aimed to summarize clinical characteristics associated with Gallium-68-prostate-specific membrane antigen (PSMA) positron emission tomography/computed tomography (^68^Ga-PSMA PET/CT) scans as patients were restaged for prostate-specific antigen (PSA) relapse after radical prostatectomy (RP) or external beam radiotherapy (EBRT). Our analyses included multiple cox regression analyses. The study evaluated 95 patients with rising values of PSAs after RP and after EBRT. Sixty 63% of patients had a positive ^68^Ga-PSMA PET/CT scan. Twelve patients (13%) had a positive site in the prostate bed, 29 patients (30%) had a positive site in the regional lymph nodes, and 19 (20%) had positive sites in distant organs. After four years follow-up, 21 patients (22%) died. Using multiple Cox regression analyses, the number of positive sites on the ^68^Ga-PSMA PET/CT scan significantly predicted overall survival (OS) (*p* = 0.0001), whereas risk score and regional locations of the positive sites were not significant in the multiple Cox regression analyses. Our study indicates that the specific findings of ^68^Ga-PSMA PET/CT scans are important because detailed findings of the scans predict the outcome after salvage treatment of patients with PSA relapse examined with ^68^Ga-PSMA PET/CT scans.

## 1. Introduction

A quarter to half of patients with prostate cancer undergoing radical prostatectomy (RP) who had an initial decline of prostate-specific antigen (PSA) to unmeasurable levels later experience a rise of PSA levels [1,2,3]. Additionally, many patients treated with external beam radiotherapy (EBRT) who demonstrate a decline of PSA levels to low nadir PSA levels later experience a rise of PSA levels [4].

With regards to patients with PSA (“biochemical”) relapse and with a long doubling time of the PSA (PSADT) >12 months before they undergo a ^68^Ga-prostate-specific membrane antigen positron emission tomography/computed tomography (^68^Ga-PSMA PET/CT) scan, and with cancers that have a low International Society of Pathology (ISUP) grade 1, few given salvage treatment progress to metastatic disease and cancer-specific death [1,2,5,6,7,8].

In contrast, many patients with PSA relapse who have a short PSADT <3 months and cancers that have a high ISUP grade 5 progress to metastatic cancer and cancer-specific death despite salvage treatment. Compared with conventional imaging modalities such as CT and bone scans, the ^68^Ga-PSMA PET/CT scan has a relatively high sensitivity, especially for patients with PSA relapse and PSA levels <2 ng/mL [9,10,11,12,13,14,15,16,17,18,19,20,21,22,23,24,25,26]. Today, the Federal Drug Administration (FDA) in the USA and many guidelines recommend that urologists and oncologists examine patients with PSA relapse with ^68^Ga-PSMA PET/CT [27,28,29,30].

Clinical characteristics before and at the imaging with ^68^Ga-PSMA PET/CT of the examined patients may be associated with the finding of positive sites [31,32,33,34,35,36,37,38]. Detection of PSMA-avid lesions is important as urologists and oncologists change the planned treatment for up to half of the patients examined with ^68^Ga-PSMA PET/CT [39,40].

However, publications on ^68^Ga-PSMA PET/CT scans differ in their reported findings. Further, most publications do not report the numbers or the regional locations of positive sites, or report whether the numbers or the regional locations of positive sites predict the overall survival (OS) for the patients. Thus, it is important to have better insight regarding the outcome following restaging with ^68^Ga-PSMA PET/CT.

Accordingly, our study of patients with PSA relapse aimed: (1) at evaluating whether clinical characteristics before or at restaging relate to findings on ^68^Ga-PSMA PET/CT; (2) at summarizing the numbers and regional locations of positive sites on ^68^Ga-PSMA PET/CT scans; and (3) at evaluating whether the number and locations of positive sites predict OS.

## 2. Material and Methods

### 2.1. Patients

Our investigation reports 95 patients with PSA relapse treated at the University of Ankara, Turkey. Our study added information to those previously published [41]. The patients had histologically/cytologically proven prostate cancer. They had undergone either EBRT or RP with or without lymph node dissection (LND), with or without adjuvant EBRT, and with or without adjuvant androgen deprivation therapy (ADT), as shown in Table 1. PSA levels (ng/mL) were determined with an ultrasensitive PSA assay and were most often reported with two decimals [42].

After RP, PSA levels typically had fallen to unmeasurable PSA levels. Similarly, after EBRT, PSA levels had typically been lowered to low nadir PSA levels. Later all patients had rising PSA levels. The rise of PSA at PSA relapse was documented in ≥3 PSA determinations carried out with ≥2 week intervals. None of the patients had had a rise of PSA due to PSA flare. The time interval between the latest PSA determination before the ^68^Ga-PSMA PET/CT and the scans was median 15 days.

### 2.2. ^68^Ga-PSMA PET/CT

The university in Ankara carried out the ^68^Ga-PSMA PET/CT scans between June 2015 and December 2018. The patients underwent ^68^Ga-DOTAGA-(1.y)fk(Sub-KuE) (^68^Ga-PSMA PET/CT) imaging and therapy (I&T) imaging as previously described [41,43]. The intravenously injected ^68^Ga activity was 111–185 MBq ^68^Ga-PSMA. Whole body ^68^Ga-PSMA PET/CT scans were obtained 45–60 min after the injection.

Attenuation-corrected PET/CT fusion images were reviewed in transaxial, coronal, and sagittal planes using Advanced Workstation Volumeshare 5 (GE Medical Systems, Houston, Pennsylvania, USA). Two experienced specialists in nuclear medicine evaluated semi-quantitatively the images according to the maximal standardized uptake values (SUV_max_) in selected sites.

The specialists in nuclear medicine considered any focal ^68^Ga-PSMA uptake higher than the background uptake that did not correspond to physiologic retention of ^68^Ga-PSMA in organs or structures as positive for the malignancy. The specialists in nuclear medicine also recorded the number of positive sites on the ^68^Ga-PSMA PET/CT scans.

### 2.3. Definitions

The aggressiveness of the prostate cancers was classified as the ISUP grade at diagnosis of the primary cancer. ISUP grade 1 corresponds to a Gleason score ≤6, ISUP grade 3 corresponds to a Gleason score 4+3 (a total of 7), ISUP grade 4 corresponds to a Gleason score 8, and ISUP grade 5 corresponds to Gleason scores 9 and 10 [44]. At RP, the estimate of the prognosis of the patients is summarized as low, intermediate, and high risk, as described previously [45].

For patients who had undergone RP and had had a decline of PSA levels after RP to unmeasurable levels, a PSA relapse was defined as rising PSA levels to >0.20 ng/mL [46]. For patients who had undergone EBRT and had had a decline of PSA levels after the EBRT to low nadir levels, PSA relapse was defined as rising PSA levels to >2 ng/mL above the nadir PSA levels [47]. Rising PSA levels were defined as rising PSA levels determined in ≥3 PSA measurements carried out with least ≥2 week intervals.

As for patients treated with RP with or without adjuvant EBRT, the relapse-free interval was the time from RP with or without adjuvant EBRT to the ^68^Ga-PSMA PET/CT. For patients treated with EBRT, the relapse-free interval was the time from end of the EBRT to the restaging ^68^Ga-PSMA PET/CT. ADT were lutein-hormone releasing hormone (LHRH) agonists and did not include third generation androgen receptor (AR) pathway inhibitors such as abiraterone, enzalutamide, apalutamide, or darolutamide.

PSADT was defined as the time estimated for a doubling of the PSA levels. PSADT was calculated based on a calculator website from the Memorial Sloan Kettering Cancer Center [48]. Previous studies of patients with PSA relapse found that those with a high ISUP grade ≥4 and a short PSADT <12 months had a high risk of death from prostate cancer [1,2,5,7].

We classified the ^68^Ga-PSMA PET/CT findings according to a PET tumor, nodes, and metastases (TNM) classification [49]. We judged the extent of the prostate cancer by the most advanced positive regional location on the ^68^Ga-PSMA PET/CT. A local positive site referred to positive sites limited to the prostate bed as PET T. Metastases in pelvic lymph nodes were positive sites in the pelvic lymph nodes with or without local positive sites as PET N.

Metastases in extra-pelvic lymph nodes were positive sites in retroperitoneal and/or supra-diaphragmatic lymph nodes, with or without positive local sites, and with or without pelvic lymph node sites as PET M1a. Bone metastases were positive sites in bones with or without positive local sites, with or without pelvic lymph node sites, and with or without extra pelvic lymph node sites as PET M1b.

Metastases in visceral organs were positive sites in the lungs, liver, or brain, with or without local sites, with or without pelvic lymph node sites, with or without extra pelvic lymph node sites, and with or without sites in bones as PET M1c.

Oligometastatic prostate cancer was defined as one to three metastatic positive sites on imaging modalities irrespective of whether the positive sites were in the prostate bed, regional lymph nodes, or in distant organs [50]. Patients with more than three positive metastatic sites were defined to have polymetastatic prostate cancer.

Clinical confirmation of the diagnosis on the ^68^Ga-PSMA PET/CT scan implied that patients with a reduction of PSA had a reduction in the positive sites and patients with a rising PSA had increasing number or size of the positive sites.

### 2.4. Salvage Treatment

Some patients with negative ^68^Ga-PSMA PET/CT scans were followed with active surveillance. Other patients were given salvage EBRT to the prostate bed. Patients with positive sites in the prostate bed were given salvage EBRT to the prostate bed. Patients with positive sites in lymph nodes or bone were treated either with chemotherapy such as docetaxel or third generation androgen receptor pathway inhibitors such as abiraterone or enzalutamide. The patients were rarely given second line systemic treatment.

### 2.5. Statistical Methods

Our study did not substitute missing data. We evaluated whether clinical characteristics had statistically significant associations with positive sites on ^68^Ga-PSMA PET/CT. The logistic regression analysis regarded positive or negative findings on the ^68^Ga-PSMA PET/CT scans as dependent variables and both categorical and continuous predictors (clinical characteristics) as independent variables. An initial full multiple logistic regression model included all potentially relevant clinical characteristics. Backwards elimination excluded variables that were insignificant in multiple logistic regression analyses.

We did not include variables that were highly correlated in the multiple logistic regression analysis, even if the variables were significant in univariate logistic regression analysis. We evaluated the significance of difference in overall survival (OS) between groups of patients with log rank tests and multiple Cox analysis tests. All statistical tests were two-sided. We considered a *p* value <0.05 as statistically significant.

We carried out the statistical analyses with use of Stata 16 (Stata corporation, College Station, TX 77845, USA).

## 3. Results

### 3.1. Initial Treatment

The 95 patients were median 68 years old, as shown in Table 1. Patients treated with RP who had ISUP grade 5 cancers especially were often given adjuvant ADT after initial RP (21 of 27 patients with ISUP grade 5 vs. 22 of 68 patients with ISUP grade ≤4, *p* <0.0005, chi2 test). Adjuvant ADT was also given to a relatively large number of patients with lymph node metastases (LNM) (16 of 23 patients with LNM vs. 27 of 72 patients without LNM, *p* = 0.007, chi2 test). The adjuvant ADT was LHRH agonists and not a combination of ADT and third-generation AR pathway inhibitors.

Patients with an ISUP grade ≥4 had shorter PSADT at PSA relapse than patients with an ISUP grade ≤3 (*p* = 0.003, Kruskal–Wallis test). The time span from the most recent PSA before the ^68^Ga-PSMA PET/CT to the scan was median 15 days.

### 3.2. ^68^Ga-PSMA PET/CT

At the time of the ^68^Ga-PSMA PET/CT, the median PSA level of the patients was 5.7 ng/mL. Six of 76 patients (9%) who had undergone RP had PSA levels before the ^68^Ga-PSMA PET/CT scan of <0.4 ng/mL. Eight of 19 patients (40%) who had been treated with EBRT had PSA levels before the ^68^Ga-PSMA PET/CT scan of <4 ng/mL.

Sixty patients (63%) had a positive ^68^Ga-PSMA PET/CT scan. These patients had 7.5 times higher median PSA levels before the ^68^Ga-PSMA PET/CT scan than the 35 patients with a negative ^68^Ga-PSMA PET/CT scan (median 11.0 vs. 1.4 ng/mL, *p* = 0.0001, Kruskal–Wallis test). Twelve patients (13%) had a positive site in the prostate bed, 29 patients (30%) had a positive site in pelvic lymph nodes, and 19 (20%) had positive sites in distant organs.

Four patients had their ^68^Ga-PSMA PET/CT findings confirmed histopathologically, whereas 91 patients had their positive findings confirmed by the clinical course after the ^68^Ga-PSMA PET/CT scan.

Adjuvant ADT was related to the ^68^Ga-PSMA PET/CT findings (*p* = 0.01, chi2 test). Patients with a short PSADT <10 months more often had a high-risk score than patients with a longer PSADT (*p* < 0.0005, Kruskal–Wallis test). In contrast, the initial RP and EBRT were not significantly related to the ^68^Ga-PSMA PET/CT findings (*p* = 0.097, chi2 test).

A third of the patients had oligometastatic prostate cancer on the ^68^Ga-PSMA PET/CT scan and another third of the patients had polymetastatic prostate cancer, as shown in Table 1.

### 3.3. Prediction of Positive Sites on ^68^Ga-PSMA PET/CT

**Figure 1:**
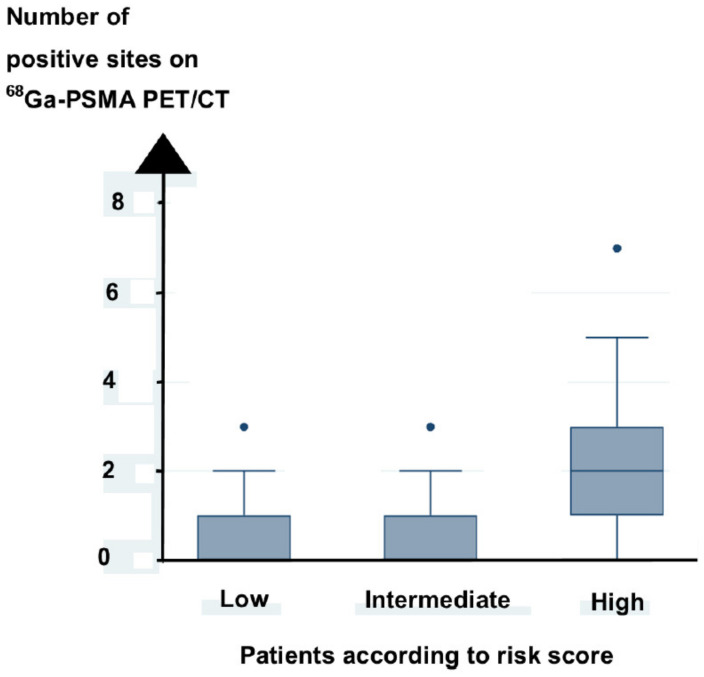
Number of positive sites on ^68^Ga-PSMA PET/CT scan increased with high risk score.

Patients with high risk at diagnosis had a higher number of positive sites on ^68^Ga-PSMA PET/CT than patients with low and intermediate risk, as shown in Figure 1.

Using multiple logistic regression analysis, PSADT was significant for positive findings with ^68^Ga-PSMA PET/CT, as shown in Table 2. In contrast, ISUP grade and risk score were significant in univariate logistic regression analyses for positive sites on the ^68^Ga-PSMA PET/CT scan, but not in multiple logistic regression analyses. Further, in univariate logistic regression analysis, many clinical characteristics were not significant for positive findings on the ^68^Ga-PSMA PET/CT scan.

At the time of the ^68^Ga-PSMA PET/CT scan patients with PSADT <3 months more often had positive sites in regional lymph nodes or distant organs than patients with PSADT >12 months (*p* < 0.0005, chi2 test). Patients with a short period from diagnosis to PSA relapse <3 years had more metastatic sites than patients with a longer period from diagnosis to PSA relapse. The PSA level up to the ^68^Ga-PSMA PET/CT had a marked impact on the ^68^Ga-PSMA PET/CT findings. Additionally, PSADT at PSA relapse had a marked impact on the ^68^Ga-PSMA PET/CT findings.

Patients with metastatic sites on the ^68^Ga-PSMA PET/CT scan had significantly more multiple positive sites than had patients with positive sites only in the prostate bed (*p* > 0.0005, chi2 test). The odds ratio (OR) for PSADT was related with positive and negative ^68^Ga-PSMA PET/CT findings (beta coefficient −0.047, *p* <0.0005, pseudo R2 = 0.37). PSA up to the ^68^Ga-PSMA PET/CT scan had a significant association with extent of regional locations of positive sites on the ^68^Ga-PSMA PET/CT scan.

### 3.4. Salvage Treatment

After ^68^Ga-PSMA PET/CT, 7 patients with negative ^68^Ga-PSMA PET/CT findings were followed with active surveillance and 23 patients were treated with salvage radiotherapy. Of patients with positive sites on the ^68^Ga-PSMA PET/CT scan, 13 were given salvage radiotherapy, 30 were given chemotherapy with docetaxel, and 17 patients were given the third-generation AR pathway inhibitors abiraterone and enzalutamide.

### 3.5. Prediction of Overall Survival

**Figure 2:**
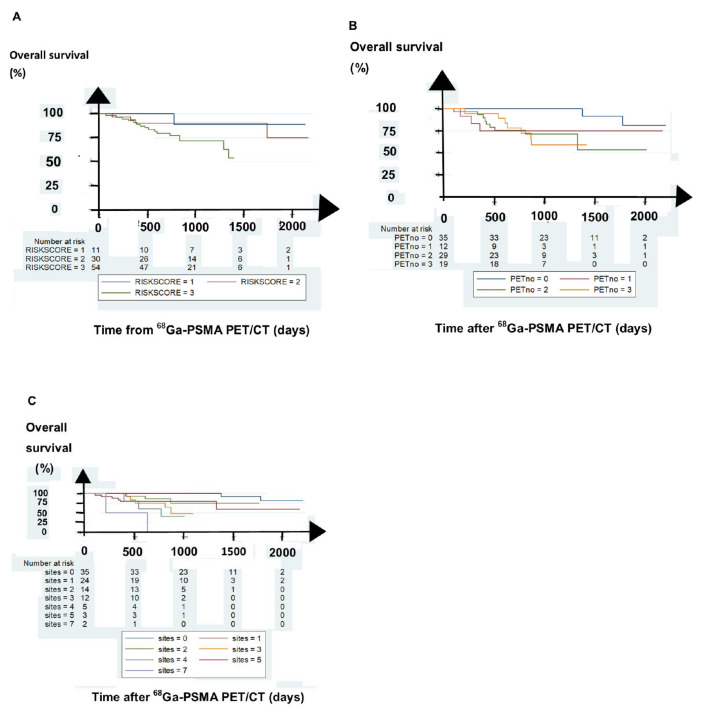
Overall survival after the 68Ga-PSMA PET/CT scan. (**A**) Shows that the risk score of the patients predicted overall survival. (**B**) Shows that the most advanced regional locations of positive sites on 68Ga-PSMA PET/CT predicted overall survival. PETno 0 denotes negative findings on 68Ga-PSMA PET/CT scans, PETno 1 denotes positive sites in the prostate bed, PETno 2 denotes positive sites in regional lymph nodes, and PETno 3 denotes positive findings in distant organs. (**C**) Shows that the number of positive sites on the 68Ga-PSMA PET/CT scan predicted overall survival.

After four years follow-up, 21 patients (22%) died. Table 3 shows the significant predictors of overall survival (OS). Risk score significantly predicted OS, as shown in Figure 2A. The most advanced regional locations of positive findings on the ^68^Ga-PSMA PET/CT scan significantly predicted OS (*p* = 0.0091, log rank test), as shown in Figure 2B. Regarding ^68^Ga-PSMA PET/CT, the number of positive sites had a significant impact on OS (*p* = 0.0001, log rank test), as shown in Figure 2C.

In multiple Cox regression analysis, only the number of positive sites on the ^68^Ga-PSMA PET/CT scan significantly predicted OS, as shown in Table 3.

## 4. Discussion

Our study showed that the regional location and number of positive sites on theh ^68^Ga-PSMA PET/CT scan had impact on the treatment of the patients with PSA relapse and predicted OS. Especially important for our study was the observation that one of three clinical characteristics for high-risk PSA relapse, ISUP grade ≥ 4 was significantly associated with finding of metastatic sites on the ^68^Ga-PSMA PET/CT scan.

**Figure 3:**
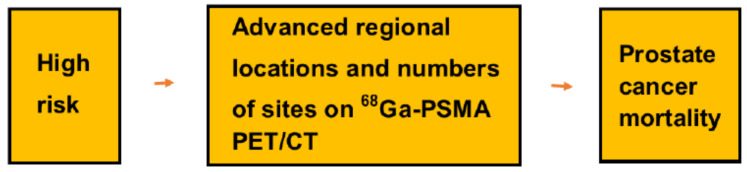
This figure summarizes the study in showing that the initial risk score and the regional locations and number of positive sites on ^68^Ga-PSMA-PET/CT scans predicted overall survival.

Further clinically important, our study showed that—in addition to high risk score—advanced regional locations and high number of positive sites on ^68^Ga-PSMA predicted impaired overall survival, a shown in Figure 3.

Francolini et al. also found that a short time to PSA relapse (2.5 years) increased the rate of positive sites in ^68^Ga-PSMA PET/CT findings [51]. Kulkarni et al. showed that patients with a high ISUP grade and a short PSADT had a high rate of ^68^Ga-PSMA PET/CT positive findings [52]. Additionally, a previous meta-analysis reported that a short PSADT at PSA relapse predicted many positive sites on ^68^Ga-PSMA PET/CT [53].

Hoffmann et al. reported that patients with PSA relapse had an S shaped relation between the PSA levels before ^68^Ga-PSMA PET/CT and the rate of positive findings on their ^68^Ga-PSMA PET/CT scans [18]. Patients with positive sites in regional lymph nodes and bones had higher median PSA values before ^68^Ga-PSMA PET/CT than those with positive sites in the prostate bed.

A key finding was that patients with a ^68^Ga-PSMA PET/CT positive site had a higher PSA level at restaging than patients with a negative finding, and that patients with a positive site in distant organs had a higher PSA level at the time of the restaging than the patients with positive sites in the prostate bed and regional lymph nodes.

Bashir et al. also showed using ^68^Ga-PSMA PET/CT that patients with PSA relapse more often had oligometastatic prostate cancer than polymetastatic prostate cancer [54]. Multiparametric MRI may better detect local relapse than ^68^Ga-PSMA PET/CT [55]. The background is that it may be difficult to differentiate between a local relapse and tracer excreted into the urinary bladder at the time of the imaging. Correspondingly, a publication of Emmett et al. indicated that patients with a negative ^68^Ga-PSMA PET/CT scan treated with salvage radiotherapy of the prostate bed had a better outcome than patients who were not treated with salvage radiotherapy [56].

Similarly, Celli et al. reported follow-up of 103 patients with negative ^68^Ga-PSMA PET/CT findings [57]. The patients were followed with active surveillance. Those with cancers of ISUP grades 4 and 5 had a relatively short median relapse-free survival (RFS) of 14 months, pointing to the risk of false negative findings with ^68^Ga-PSMA PET/CT.

Artibani et al. underlined that it is clinically important to salvage treatment in the phase of early PSA relapse and to distinguish between local and metastatic sites at PSA relapse [58]. Conventional imaging modalities do not allow the categorization of the recurrent site for patients with PSA relapse and PSA levels below 2 ng/mL. Restaging with ^68^Ga-PSMA PET/CT is crucial for decisions regarding the most relevant treatments for patients with early PSA relapse.

Another publication with three years of follow-up after ^68^Ga-PSMA PET/CT showed that 65% of patients with negative sites or positive sites only in the prostate bed had a three-year progression-free survival (PFS), whereas this number was lower (45%) for patients with positive sites outside the prostate bed [58]. The study underlines that the regional location of positive sites on restaging ^68^Ga-PSMA PET/CT has prognostic significance.

**Figure 4:**
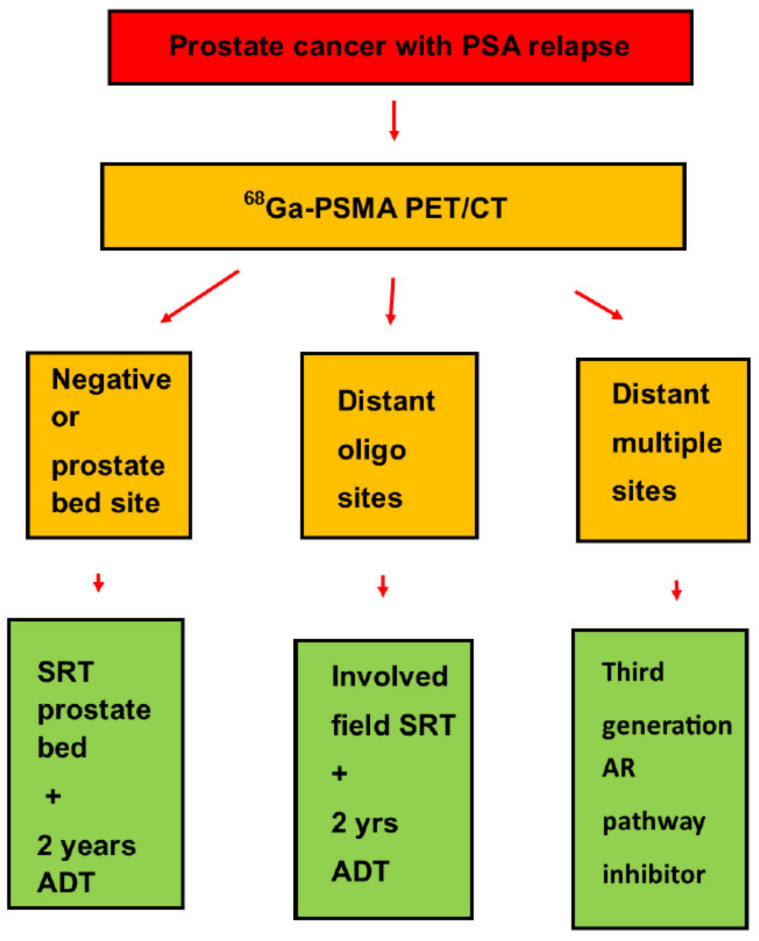
This figure summarizes how ^68^Ga-PSMA PET/CT findings may change treatment decisions for the salvage treatment of patients with PSA relapse

Further another previous publication showed that patients who were given salvage radiotherapy to the prostate bed when they had extremely low pretreatment PSA most often did not develop a new recurrence [59]. Previous publications reported salvage treatment for patients with PSA relapse based on ^68^Ga-PSMA PET/CT, as indicated in Figure 4. For up to two thirds of the patients with PSA relapse, treatment based on ^68^Ga-PSMA PET/CT differed from the treatment plans based on only conventional imaging modalities [60,61].

Our study provides relevant correlations with others regarding non-metastatic prostate cancer. Three large clinical trials used PSA >2 ng/mL and PSADT <10 months as inclusion criteria and showed a benefit when AR pathways inhibitors, enzalutamide (PROSPER trial), apalutamide (SPARTAN trial), and darolutamide was added to conventional ADT [62,63,64]. Importantly, our patients had the same range of pre-test PSA levels as had patients reported in the three trials of patients with non-metastatic prostate cancer. A previous study [65] like our study points to ^68^Ga-PSMA PET/CT detecting metastatic sites.

Our study also provides relevant correlations with others regarding the concept of oligometastatic prostate cancer. The stage is defined due to the findings with second generation imaging modalities such as choline and PSMA PET/CT. Some authors have used the information from the PET/CT scans to target the positive sites with precision EBRT with the aim to delay systemic treatment [66,67,68,69]. The overall survival with this approach has not been encouraging [70,71] compared with the results from systemic treatment with combined ADT and second-generation AR pathway inhibitors [62,63,64].

To the best of our knowledge, the prediction of outcome from number of positive sites on ^68^Ga-PSMA PET/CT scans for patients with PSA relapse has not been reported before. The finding can be validated in a complementary ongoing multicenter study.

Ongoing trials further are being undertaken evaluate the role of ^68^Ga-PSMA PET/CT in treatment decision-making processes [72]. Future trials may investigate whether another approach to patients with PSA relapse combining targeted radiotherapy and systemic treatment might improve OS.

Our study had limitations. ^68^Ga-PSMA PET/CT might have given some false negative findings for the prostate bed. The scans may also have given some false negative findings for pelvic lymph nodes due to undetected micrometastases with tumor diameters <5 mm [71,72]. Our study did not include initial staging at the initial treatment with ^68^Ga-PSMA PET/CT or the staging of patients with PSA relapse with ^18^F-PSMA PET/CT scans [73]. Our study did not evaluate PSMA PET/MRI, an alternative imaging modality that avoids the radioactivity exposure of the CT component of the PET/CT scans [74].

## 5. Conclusions

Most patients with PSA relapse after RP or EBRT have positive findings with ^68^Ga-PSMA PET/CT. The positive findings had implications for the salvage treatment and predicted overall survival.

## 6. Ethical Approval

All patients had given informed consent to undergo ^68^Ga-PSMA PET/CT and for their findings to be evaluated and published. The Science-Ethics committee of the University of Ankara, Turkey, approved the present analyses of the study group of patients as of 4 March 2021 (approval number 12-171-21).

## Figures and Tables

**Table 1:** Patient characteristics.

Patient Characteristic		No pts	MedianValue	Lower and Upper IQ	Total Range
		95			
Age (years)			68	64–72	55–87
Initial TNM staging	T1	6			
	T2	53			
	T3	18			
	N0	72			
	N1	23			
ISUP score	1	9			
	2–3	32			
	4	27			
	5	26			
Risk	Low	11			
	Intermediate	10			
	High	54			
Initial treatment	RP	45			
	RP and adjuvant ADT	49			
	RP and adjuvant EBRT	14			
	EBRT				
Relapse-free interval until PSA relapse (mos)			24	13–40	4–156
PSADT (mos)			10.6	4.5–48	1–168
Time from the latest PSA before the ^68^Ga-PSMA PET/CT to the scan			15	11–19	6–27
PSA at ^68^Ga-PSMA PET/CT (ng/mL)			5.7	1.2–12.6	0.10–155.6
^68^Ga-PSMA PET/CT sites	negative		35		
	Prostate bed	12			
	Regional lymph nodes	29			
	Distant organs	19			
Number of positive sites on ^68^Ga-PSMA PET/CT	0	35			
	1	24			
	2	13			
	3	12			
	4	5			
	5	3			
	7	2			
Treatment after the ^68^Ga-PSMA PET/CT	Active surveillance	7			
	Salvage radiotherapy	36			
	Docetaxel	31			
	Abiraterone/Enzalutamide	21			

Abbreviations: ADT = androgen deprivation therapy, EBRT = external beam radiotherapy, ISUP = International Society of Urologic Pathology, IQ = interquartile, mos = months, no = number, pts = patients, PSADT = prostate-specific antigen doubling time, RP = radical prostatectomy, TNM = tumor, nodes, and metastases.

**Table 2:** Clinical characteristics and association with positive ^68^Ga-PSMA PET/CT scans (*n* = 95).

Clinical Characteristics	Prediction in Logistic Regression Analysis	
	Univariate analysis	Multivariate analysis
Age	NS	NS
N status	NS	NS
ISUP score	0.005	NS
Risk score	<0.005	NS
Number of positive sites	NS	NS
Initial treatment (RP vs. EBRT)	NS	NS
Adjuvant ADT	0.002	NS
Disease free interval	NS	NS
Lymph node metastases at initial treatment	NS	NS
PSADT	<0.0005	<0.0005
PSA at ^68^Ga-PSMA PET/CT	0.010	NS

Abbreviations as in Table 1. NS = not significant.

**Table 3:** Analyses of overall survival.

Predictive Factors	Cox Regression Analysis	
	Univariate	Multiple
ISUP score	NS	-
Risk score	0.039	NS
Initial treatment	NS	-
Adjuvant ADT	NS	-
Interval from initial treatment to PSA recurrence	NS	-
PSA at ^68^Ga-PSMA PET/CT	NS	-
PSADT	NS	-
Most advanced regional location at ^68^Ga PSMA PET/CT	0.004	NS
No of sites on ^68^Ga-PSMA PET/CT	0.0001	0.0001

## Data Availability

The database for the study is not public available.

## References

[1] Antonarakis E.S., Feng Z., Trock B.J., Humphreys E.B., Carducci M.A., Partin A.W., Walsh P.C., Eisenberger M.A. (2012). The natural history of metastatic progression in men with prostate-specific antigen recurrence after radical prostatectomy: Long-term follow-up. BJU Int..

[2] Freedland S.J., Humphreys E.B., Mangold L.A., Eisenberger M., Dorey F.J., Walsh P.C., Partin A.W. (2005). Risk of prostate cancer-specific mortality following biochemical recurrence after radical prostatectomy. JAMA.

[3] Paller C.J., Antonarakis E.S. (2013). Management of biochemically recurrent prostate cancer after local therapy: Evolving standards of care and new directions. Clin. Adv. Hematol. Oncol..

[4] Kestin L.L., Vicini F.A., Ziaja E.L., Stromberg J.S., Frazier R.C., Martinez A.A. (1999). Defining biochemical cure for prostate carcinoma patients treated with external beam radiation therapy. Cancer.

[5] D’Amico A.V., Moul J., Carroll P.R., Sun L., Lubeck D., Chen M.H. (2004). Prostate specific antigen doubling time as a surrogate end point for prostate cancer specific mortality following radical prostatectomy or radiation therapy. J. Urol..

[6] Denham J.W., Steigler A., Wilcox C., Lamb D.S., Joseph D., Atkinson C., Matthews J., Tai K.H., Spry N.A., Christie D. (2008). Time to biochemical failure and prostate-specific antigen doubling time as surrogates for prostate cancer-specific mortality: Evidence from the TROG 96.01 randomised controlled trial. Lancet Oncol..

[7] Brockman J.A., Alanee S., Vickers A.J., Scardino P.T., Wood D.P., Kibel A.S., Lin D.W., Bianco F.J., Rabah D.M., Klein E.A. (2015). Nomogram predicting prostate cancer-specific mortality for men with biochemical recurrence after radical prostatectomy. Eur. Urol..

[8] Zumsteg Z.S., Spratt D.E., Romesser P.B., Pei X., Zhang Z., Polkinghorn W., McBride S., Kollmeier M., Yamada Y., Zelefsky M.J. (2015). The natural history and predictors of outcome following biochemical relapse in the dose escalation era for prostate cancer patients undergoing definitive external beam radiotherapy. Eur. Urol..

[9] Afshar-Oromieh A., Avtzi E., Giesel F.L., Holland-Letz T., Linhart H.G., Eder M., Eisenhut M., Boxler S., Hadaschik B.A., Kratochwil C. (2015). The diagnostic value of PET/CT imaging with the ^68^Ga-labelled PSMA ligand HBED-CC in the diagnosis of recurrent prostate cancer. Eur. J. Nucl. Med. Mol. Imaging.

[10] Eiber M., Maurer T., Souvatzoglou M., Beer A.J., Ruffani A., Haller B., Graner F.P., Kubler H., Haberhorn U., Eisenhut M. (2015). Evaluation of hybrid ^68^Ga-PSMA ligand PET/CT in 248 patients with biochemical recurrence after rtadical prostatectomy. J. Nucl. Med..

[11] Morigi J.J., Stricker P.D., van Leeuwen P.J., Tang R., Ho B., Nguyen Q., Hruby G., Fogarty G., Jagavkar R., Kneebone A. (2015). Prospective comparison of 18F-Fluoromethylcholine versus ^68^Ga-PSMA PET/CT in prostate cancer patients who have rising PSA after curative treatment and are being considered for targeted therapy. J. Nucl. Med..

[12] van Leeuwen P.J., Stricker P., Hruby G., Kneebone A., Ting F., Thompson B., Nguyen Q., Ho B., Emmett L. (2016). ^68^Ga-PSMA has a high detection rate of prostate cancer recurrence outside the prostatic fossa in patients being considered for salvage radiation treatment. BJU Int..

[13] Verburg F.A., Pfister D., Heidenreich A., Vogg A., Drude N.I., Voo S., Mottaghy F.M., Behrendt F.F. (2016). Extent of disease in recurrent prostate cancer determined by [^68^Ga] PSMA-HBED-CC PET/CT in relation to PSA levels, PSA doubling time and Gleason score. Eur. J. Nucl. Med. Mol. Imaging.

[14] Afshar-Oromieh A., Holland-Letz T., Giesel F.L., Kratochwil C., Mier W., Haufe S., Debus N., Eder M., Eisenhut M., Schafer M. (2017). Diagnostic performance of ^68^Ga-PSMA-11 (HBED-CC) PET/CT in patients with recurrent prostate cancer: Evaluation in 1007 patients. Eur. J. Nucl. Med. Mol. Imaging.

[15] Freitag M.T., Radtke J.P., Afshar-Oromieh A., Roethke M.C., Hadaschik B.A., Gleave M., Bonekamp D., Kopka K., Eder M., Heusser T. (2017). Local recurrence of prostate cancer after radical prostatectomy is at risk to be missed in ^68^Ga-PSMA-11-PET of PET/CT and PET/MRI: Comparison with mpMRI integrated in simultaneous PET/MRI. Eur. J. Nucl. Med. Mol. Imaging.

[16] Eissa A., Elsherbiny A., Coelho R.F., Rassweiler J., Davis J.W., Porpiglia F., Patel V.R., Prandini N., Micali S., Sighinolfi M.C. (2018). The role of ^68^Ga-PSMA PET/CT scan in biochemical recurrence after primary treatment for prostate cancer: A systematic review of the literature. Minerva Urol. Nefrol..

[17] Boreta L., Gadzinski A.J., Wu S.Y., Xu M., Greene K., Quanstrom K., Nguyen H.G., Carroll P.R., Hope T.A., Feng F.Y. (2019). Location of recurrence by Gallium-68 PSMA-11 PET scan in prostate cancer patients eligible for salvage radiotherapy. Urology.

[18] Hoffmann M.A., Buchholz H.G., Wieler H.J., Hofner T., Muller-Hubenthal J., Trampert L., Schreckenberger M. (2019). The positivity rate of ^68^Gallium-PSMA-11 ligand PET/CT depends on the serum PSA-value in patients with biochemical recurrence of prostate cancer. Oncotarget.

[19] McCarthy M., Francis R., Tang C., Watts J., Campbell A. (2019). A multicenter prospective clinical trial of ^68^Gallium PSMA HBED-CC PET-CT restaging in biochemically relapsed prostate carcinoma: Oligometastatic rate and distribution compared with standard imaging. Int. J. Radiat. Oncol. Biol. Phys..

[20] Schwenck J., Olthof S.C., Pfannenberg C., Reischl G., Wegener D., Marzec J., Bedke J., Stenzl A., Nikolaou K., la Fougere C. (2019). Intention-to-treat analysis of ^68^Ga-PSMA and (11)C-Choline PET/CT versus CT for prostate cancer recurrence after surgery. J. Nucl. Med..

[21] Joshi A., Roberts M.J., Perera M., Williams E., Rhee H., Pryor D., Lehman M., Heathcote P., Wood S., Coucher J. (2020). The clinical efficacy of PSMA PET/MRI in biochemically recurrent prostate cancer compared with standard of care imaging modalities and confirmatory histopathology: Results of a single-centre, prospective clinical trial. Clin. Exp. Metastasis.

[22] Ribeiro A.M.B., Lima E.N.P., Zequi S.C. (2021). Evaluation of the clinical use of PET/CT with ^68^Ga-PSMA for the assessment of biochemical recurrence of low or intermediate-risk prostate cancer. Urol. Oncol..

[23] Tseng J.R., Yu K.J., Liu F.Y., Yang L.Y., Hong J.H., Yen T.C., Pang S.T., Wang L.J. (2021). Comparison between ^68^Ga-PSMA-11 PET/CT and multiparametric magnetic resonance imaging in patients with biochemically recurrent prostate cancer following robot-assisted radical prostatectomy. J. Formos. Med. Assoc..

[24] Pienta K.J., Gorin M.A., Rowe S.P., Carroll P.R., Pouliot F., Probst S., Saperstein L., Preston M.A., Alva A.S., Patnaik A. (2021). A Phase 2/3 Prospective multicenter study of the diagnostic accuracy of prostate-specific membrane antigen PET/CT with ^18^F-DCFPyL in prostate cancer patients (OSPREY). J. Urol..

[25] Morris M.J., Rowe S.P., Gorin M.A., Saperstein L., Pouliot F., Josephson D.Y., Wong J.Y., Pantel A.R., Cho S.Y., Gage K.L. (2021). Diagnostic performance of ^18^F-DCFPyL-PET/CT in men with biochemically recurrent prostate cancer: Results from the CONDOR phase 3, multicenter study. Clin. Cancer Res..

[26] Afshar-Oromieh A., da Cunha M.L., Wagner J., Haberkorn U., Debus N., Weber W., Eiber M., Holland-Letz T., Rauscher I. (2021). Performance of [^68^Ga] Ga-PSMA-11 PET/CT in patients with recurrent prostate cancer after prostatectomy-a multi-centre evaluation of 2533 patients. Eur. J. Nucl. Med. Mol. Imaging.

[27] DAPROCA (2020). [Imaging of Prostate Cancer v 2.0] (Billiediagnostik ved Prostatacancer Version 2.0). www.daproca:billeddiagnostik_v2.0_pdf.

[28] Cornford P., van den Bergh R.C.N., Briers E., Van den Broeck T., Cumberbatch M.G., De Santis M., Fanti S., Fossati N., Gandaglia G., Gillessen S. (2021). EAU-EANM-ESTRO-ESUR-SIOG guidelines on prostate cancer. Part II-2020 update: Treatment of relapsing and metastatic prostate cancer. Eur. Urol..

[29] Gandaglia G., Leni R., Fossati N., Cucchiara V., Montorsi F., Briganti A. (2021). Prostate-specific membrane antigen imaging in clinical guidelines: European Association of Urology, National Comprehensive Cancer Network, and beyond. Eur. Urol. Focus.

[30] FDA FDA Approves First PSMA-Targeted PET Imaging Drug for Men with Prostate Cancer.

[31] Cantiello F., Crocerossa F., Russo G.I., Gangemi V., Ferro M., Vartolomei M.D., Lucarelli G., Mirabelli M., Scafuro C., Ucciero G. (2018). Comparison between ^64^Cu-PSMA-617 PET/CT and ^18^F-Choline PET/CT imaging in early diagnosis of prostate cancer biochemical recurrence. Clin. Genitourin. Cancer.

[32] Rauscher I., Duwel C., Haller B., Rischpler C., Heck M.M., Gschwend J.E., Schwaiger M., Maurer T., Eiber M. (2018). Efficacy, predictive factors, and prediction nomograms for ^68^Ga-labeled prostate-specific membrane antigen-ligand positron-emission tomography/computed tomography in early biochemical recurrent prostate cancer after radical prostatectomy. Eur. Urol..

[33] Akdemir E.N., Tuncel M., Akyol F., Bilen C.Y., Baydar D.E., Karabulut E., Ozen H., Caglar M. (2019). ^68^Ga-labelled PSMA ligand HBED-CC PET/CT imaging in patients with recurrent prostate cancer. World J. Urol..

[34] Calais J., Ceci F., Eiber M., Hope T.A., Hofman M.S., Rischpler C., Bach-Gansmo T., Nanni C., Savir-Baruch B., Elashoff D. (2019). ^18^F-fluciclovine PET-CT and ^68^Ga-PSMA-11 PET-CT in patients with early biochemical recurrence after prostatectomy: A prospective, single-centre, single-arm, comparative imaging trial. Lancet Oncol..

[35] Bianchi L., Borghesi M., Schiavina R., Castellucci P., Ercolino A., Bianchi F.M., Barbaresi U., Polverari G., Brunocilla E., Fanti S. (2020). Predictive accuracy and clinical benefit of a nomogram aimed to predict ^68^Ga-PSMA PET/CT positivity in patients with prostate cancer recurrence and PSA <1 ng/mL external validation on a single institution database. Eur. J. Nucl. Med. Mol. Imaging.

[36] Ceci F., Bianchi L., Borghesi M., Polverari G., Farolfi A., Briganti A., Schiavina R., Brunocilla E., Castellucci P., Fanti S. (2020). Prediction nomogram for ^68^Ga-PSMA-11 PET/CT in different clinical settings of PSA failure after radical treatment for prostate cancer. Eur. J. Nucl. Med. Mol. Imaging.

[37] Luiting H.B., van Leeuwen P.J., Remmers S., Donswijk M., Busstra M.B., Bakker I.L., Brabander T., Stokkel M., van der Poel H.G., Roobol M.J. (2020). Optimal timing of prostate specific membrane antigen positron emission tomography/computerized tomography for biochemical recurrence after radical prostatectomy. J. Urol..

[38] Sartor O., Hope T.A., Calais J., Fendler W.P. (2021). Oliver Sartor Talks with Thomas A. Hope, Jeremie Calais, and Wolfgang P. Fendler about FDA approval of PSMA. J. Nucl. Med..

[39] Fendler W.P., Ferdinandus J., Czernin J., Eiber M., Flavell R.R., Behr S.C., Wu I.K., Lawhn-Heath C., Pampaloni M.H., Reiter R.E. (2020). Impact of ^68^Ga-PSMA-11 PET on the management of recurrent prostate cancer in a prospective single-arm clinical trial. J. Nucl. Med..

[40] Sonni I., Eiber M., Fendler W.P., Alano R.M., Vangala S.S., Kishan A.U., Nickols N., Rettig M.B., Reiter R.E., Czernin J. (2020). Impact of ^68^Ga-PSMA-11 PET/CT on staging and management of prostate cancer patients in various clinical settings: A prospective single-center Study. J. Nucl. Med..

[41] Soydal C., Urun Y., Suer E., Nak D., Ozkan E., Kucuk N.O. (2020). Predictor of ^68^Ga PSMA PET/CT positivity in patients with prostate cancer. Q. J. Nucl. Med. Mol. Imaging.

[42] Grivas N., de Bruin D., Barwari K., van Muilekom E., Tillier C., van Leeuwen P.J., Wit E., Kroese W., van der Poel H. (2019). Ultrasensitive prostate-specific antigen level as a predictor of biochemical progression after robot-assisted radical prostatectomy: Towards risk adapted follow-up. J. Clin. Lab. Anal..

[43] Weineisen M., Schottelius M., Simecek J., Baum R.P., Yildiz A., Beykan S., Kulkarni H.R., Lassmann M., Klette I., Eiber M. (2015). ^68^Ga- and 177Lu-Labeled PSMA I&T: Optimization of a PSMA-targeted theranostic concept and first proof-of-concept Human Studies. J. Nucl. Med..

[44] Epstein J.I., Egevad L., Amin M.B., Delahunt B., Srigley J.R., Humphrey P.A., Grading C. (2016). The 2014 International Society of Urological Pathology (ISUP) Consensus Conference on Gleason grading of prostatic carcinoma: Definition of grading patterns and proposal for a new grading system. Am. J. Surg. Pathol..

[45] D’Amico A.V., Whittington R., Malkowicz S.B., Schultz D., Blank K., Broderick G.A., Tomaszewski J.E., Renshaw A.A., Kaplan I., Beard C.J. (1998). Biochemical outcome after radical prostatectomy, external beam radiation therapy, or interstitial radiation therapy for clinically localized prostate cancer. JAMA.

[46] Cookson M.S., Aus G., Burnett A.L., Canby-Hagino E.D., D’Amico A.V., Dmochowski R.R., Eton D.T., Forman J.D., Goldenberg S.L., Hernandez J. (2007). Variation in the definition of biochemical recurrence in patients treated for localized prostate cancer: The American Urological Association prostate guidelines for localized prostate cancer update panel report and recommendations for a standard in the reporting of surgical outcomes. J. Urol..

[47] Roach M., Hanks G., Thames H., Schellhammer P., Shipley W.U., Sokol G.H., Sandler H. (2006). Defining biochemical failure following radiotherapy with or without hormonal therapy in men with clinically localized prostate cancer: Recommendations of the RTOG-ASTRO Phoenix Consensus Conference. Int. J. Radiat. Oncol. Biol. Phys..

[48] (2010). Memorial Sloan Kettering Cancer Center. https://www.mskcc.org/nomograms/prostate/psa_doubling_time.

[49] Eiber M., Herrmann K., Calais J., Hadaschik B., Giesel F.L., Hartenbach M., Hope T., Reiter R., Maurer T., Weber W.A. (2018). Prostate cancer molecular imaging standardized evaluation (PROMISE): Proposed miTNM classification for the interpretation of PSMA-ligand PET/CT. J. Nucl. Med..

[50] Lecouvet F.E., Oprea-Lager D.E., Liu Y., Ost P., Bidaut L., Collette L., Deroose C.M., Goffin K., Herrmann K., Hoekstra O.S. (2018). Use of modern imaging methods to facilitate trials of metastasis-directed therapy for oligometastatic disease in prostate cancer: A consensus recommendation from the EORTC Imaging Group. Lancet Oncol..

[51] Francolini G., Detti B., Bottero M., Zilli T., Lancia A., Bruni A., Borghesi S., Mariotti M., Castellucci P., Fanti S. (2021). Detection rate, pattern of relapse and influence on therapeutic decision of PSMA PET/CT in patients affected by biochemical recurrence after radical prostatectomy, a retrospective case series. Clin. Transl. Oncol..

[52] Kulkarni M., Hughes S., Mallia A., Gibson V., Young J., Aggarwal A., Morris S., Challacombe B., Popert R., Brown C. (2020). The management impact of ^68^gallium-tris(hydroxypyridinone) prostate-specific membrane antigen (^68^Ga-THP-PSMA) PET-CT imaging for high-risk and biochemically recurrent prostate cancer. Eur. J. Nucl. Med. Mol. Imaging.

[53] Pereira Mestre R., Treglia G., Ferrari M., Pascale M., Mazzara C., Azinwi N.C., Llado A., Stathis A., Giovanella L., Roggero E. (2019). Correlation between PSA kinetics and PSMA-PET in prostate cancer restaging: A meta-analysis. Eur. J. Clin. Investig..

[54] Bashir U., Tree A., Mayer E., Levine D., Parker C., Dearnaley D., Oyen W.J.G. (2019). Impact of Ga-68-PSMA PET/CT on management in prostate cancer patients with very early biochemical recurrence after radical prostatectomy. Eur. J. Nucl. Med. Mol. Imaging.

[55] Tulsyan S., Das C.J., Tripathi M., Seth A., Kumar R., Bal C. (2017). Comparison of ^68^Ga-PSMA PET/CT and multiparametric MRI for staging of high-risk prostate cancer^68^Ga-PSMA PET and MRI in prostate cancer. Nucl. Med. Commun..

[56] Emmett L., van Leeuwen P.J., Nandurkar R., Scheltema M.J., Cusick T., Hruby G., Kneebone A., Eade T., Fogarty G., Jagavkar R. (2017). Treatment outcomes from ^68^Ga-PSMA PET/CT-informed salvage radiation treatment in men with rising PSA after radical prostatectomy: Prognostic value of a negative PSMA PET. J. Nucl. Med..

[57] Celli M., De Giorgi U., Caroli P., Di Iorio V., Fantini L., Rossetti V., Foca F., Nicolini S., Giganti M., Paganelli G. (2021). Clinical value of negative ^68^Ga-PSMA PET/CT in the management of biochemical recurrent prostate cancer patients. Eur. J. Nucl. Med. Mol. Imaging.

[58] Artibani W., Porcaro A.B., De Marco V., Cerruto M.A., Siracusano S. (2018). Management of biochemical recurrence after primary curative treatment for prostate cancer: A review. Urol. Int..

[59] Emmett L., Tang R., Nandurkar R., Hruby G., Roach P., Watts J.A., Cusick T., Kneebone A., Ho B., Chan L. (2020). 3-year freedom from progression after ^68^Ga-PSMA PET/CT-triaged management in men with biochemical recurrence after radical prostatectomy: Results of a prospective multicenter trial. J. Nucl. Med..

[60] Stish B.J., Pisansky T.M., Harmsen W.S., Davis B.J., Tzou K.S., Choo R., Buskirk S.J. (2016). Improved metastasis-free and survival outcomes with early salvage radiotherapy in men with detectable prostate-specific antigen after prostatectomy for prostate cancer. J. Clin. Oncol..

[61] Roach P.J., Francis R., Emmett L., Hsiao E., Kneebone A., Hruby G., Eade T., Nguyen Q.A., Thompson B.D., Cusick T. (2018). The impact of ^68^Ga-PSMA PET/CT on management intent in prostate cancer: Results of an australian prospective multicenter study. J. Nucl. Med..

[62] Hussain M., Fizazi K., Saad F., Rathenborg P., Shore N., Ferreira U., Ivashchenko P., Demirhan E., Modelska K., De Phung B.S. (2018). Enzalutamide in men with nonmetastatic castration-resistant prostate cancer. N. Engl. J. Med..

[63] Smith M.R., Saad F., Chowdhury S., Oudard S., Hadaschik B.A., Graff J.N., Olmos D., Mainwaring P.N., Lee J.Y., Uemura H. (2018). Apalutamide treatment and metastasis-free survival in prostate cancer. N. Engl. J. Med..

[64] Fizazi K., Shore N., Tammela T.L., Ulys A., Vjaters E., Polyakov S., Jievaltas M., Luz M., Alekseev B., Kuss I. (2019). Darolutamide in nonmetastatic, castration-resistant prostate cancer. N. Engl. J. Med..

[65] Wang B., Liu C., Wei Y., Meng J., Zhang Y., Gan H., Xu X., Wan F., Pan J., Ma X. (2020). A Prospective trial of ^68^Ga-PSMA and ^18^F-FDG PET/CT in nonmetastatic prostate cancer patients with an early PSA progression during castration. Clin. Cancer Res..

[66] Ost P., Reynders D., Decaestecker K., Fonteyne V., Lumen N., De Bruycker A., Lambert B., Delrue L., Bultijnck R., Claeys T. (2018). Surveillance or metastasis-directed therapy for oligometastatic prostate cancer recurrence: A prospective, randomized, multicenter phase II trial. J. Clin. Oncol..

[67] Phillips R., Shi W.Y., Deek M., Radwan N., Lim S.J., Antonarakis E.S., Rowe S.P., Ross A.E., Gorin M.A., Deville C. (2020). Outcomes of observation vs stereotactic ablative radiation for oligometastatic prostate cancer: The ORIOLE Phase 2 randomized clinical trial. JAMA Oncol..

[68] Kneebone A., Hruby G., Ainsworth H., Byrne K., Brown C., Guo L., Guminski A., Eade T. (2018). Stereotactic body radiotherapy for oligometastatic prostate cancer detected via prostate-specific membrane antigen positron emission tomography. Eur. Urol. Oncol..

[69] Vale C.L., Fisher D., Kneebone A., Parker C., Pearse M., Richaud P., Sargos P., Sydes M.R., Brawley C., Brihoum M. (2020). Adjuvant or early salvage radiotherapy for the treatment of localised and locally advanced prostate cancer: A prospectively planned systematic review and meta-analysis of aggregate data. Lancet.

[70] Jansen B.H.E., Bodar Y.J.L., Zwezerijnen G.J.C., Meijer D., van der Voorn J.P., Nieuwenhuijzen J.A., Wondergem M., Roeleveld T.A., Boellaard R., Hoekstra O.S. (2021). Pelvic lymph-node staging with ^18^F-DCFPyL PET/CT prior to extended pelvic lymph-node dissection in primary prostate cancer—The SALT trial. Eur. J. Nucl. Med. Mol. Imaging.

[71] Kopp J., Kopp D., Bernhardt E., Manka L., Beck A., Gerullis H., Karakiewicz P., Schoerner W., Hammerer P., Schiffmann J. (2020). ^68^Ga-PSMA PET/CT based primary staging and histological correlation after extended pelvic lymph node dissection at radical prostatectomy. World J. Urol..

[72] Calais J., Czernin J., Fendler W.P., Elashoff D., Nickols N.G. (2019). Randomized prospective phase III trial of ^68^Ga-PSMA-11 PET/CT molecular imaging for prostate cancer salvage radiotherapy planning [PSMA-SRT]. BMC Cancer.

[73] Rousseau E., Wilson D., Lacroix-Poisson F., Krauze A., Chi K., Gleave M., McKenzie M., Tyldesley S., Goldenberg S.L., Benard F. (2019). A prospective study on ^18^F-DCFPyL PSMA PET/CT imaging in biochemical recurrence of prostate cancer. J. Nucl. Med..

[74] Evangelista L., Zattoni F., Cassarino G., Artioli P., Cecchin D., Dal Moro F., Zucchetta P. (2020). PET/MRI in prostate cancer: A systematic review and meta-analysis. Eur. J. Nucl. Med. Mol. Imaging.

